# Double viral vector technology for selective manipulation of neural pathways with higher level of efficiency and safety

**DOI:** 10.1038/s41434-020-00212-y

**Published:** 2021-01-11

**Authors:** Yoshinori Koshimizu, Kaoru Isa, Kenta Kobayashi, Tadashi Isa

**Affiliations:** 1 Division of Physiology and Neurobiology, Department of Neuroscience, Graduate School of Medicine, Kyoto University, Kyoto, Japan; 2 Core Research for Evolutionary Science and Technology, Japan Science and Technology Agency, Tokyo, Japan; 3 Section of Viral Vector Development, National Institute of Physiological Sciences, Okazaki, Japan; 4 SOKENDAI (The Graduate University of Advanced Studies), Hayama, Japan; 5 Human Brain Research Center, Graduated School of Medicine, Kyoto University, Kyoto, Japan; 6 Institute for the Advanced Study of Human Biology (WPI-ASHBi), Kyoto University, Kyoto, Japan

**Keywords:** Neuroscience, Transfection

## Abstract

Pathway-selective gene delivery would be critical for future gene therapy against neuropsychiatric disorders, traumatic neuronal injuries, or neurodegenerative diseases, because the impaired functions depend on neural circuits affected by the insults. Pathway-selective gene delivery can be achieved by double viral vector techniques, which combine an injection of a retrograde transport viral vector into the projection area of the target neurons and that of an anterograde viral vector into their somas. In this study, we tested the efficiency of gene delivery with different combinations of viral vectors to the pathway extending from the ventral tegmental area (VTA) to the cortical motor regions in rats, considered to be critical in the promotion of motor recovery from neural injuries. It was found that retrograde recombinant adeno-associated virus 2-retro (rAAV2reto) combined with anterograde AAVDJ (type2/type4/type5/type8/type9/avian/bovine/caprine chimera) exhibited the highest transduction efficiency in the short term (3–6 weeks) but high toxicity in the long term (3 months). In contrast, the same rAAV2reto combined with anterograde AAV5 displayed moderate transduction efficiency in the short term but low toxicity in the long term. These data suggest that the combination of anterograde AAV5 and retrograde rAAV2retro is suitable for safe and efficient gene delivery to the VTA-cortical pathway.

## Introduction

Viral vectors are powerful tools for gene therapy and have been applied against neurodegenerative diseases, such as Alzheimer’s and Parkinson’s diseases (PD) [1, 2]. Most of the current methods are nonselective to cell types around the injection site. Viral vectors infect all cell types including both excitatory and inhibitory neurons and glial cells as long as nonselective promotors such as the cytomegalovirus promotor are applied [3]. To avoid side effects and enhance efficiency, it is desirable to make the transduction cell-type specific. One way is to use cell-type specific promotors such as the neuron-specific synapsin promotor or the excitatory neuron-specific CaMKII promotor [4–6]. Another way to enable cell-type specificity is to make the transduction pathway-selective. For future gene therapy, such pathway-selective gene delivery may be beneficial, as some neuropsychiatric disorders and neuronal injuries depend on particular neural pathways, such as addiction on the mesoaccumbal pathway, PD on the nigrostriatal pathways and motor paralysis on the corticospinal and other descending motor pathways [7–9]. In mice, which can be transgenic, combining the Cre-lines for specific promotors to the target cell type and viral vectors with lox-P sequence enables pathway specificity if the promotor is known [10]. However, for application in humans or nonhuman primates, double vector technologies would be realistic in which a retrograde transport viral vector is injected in the target area of the cells in question, and another anterograde transport vector is injected at the location of their cell somas, combined with the regulatory system of gene expression such as Cre or Tet [11, 12]. However, this technique is still new, and the process of selecting the vectors and serotypes is not well established. Thus, it is not obvious whether the efficiency and safety of gene transduction of individual vectors with single use can explain the efficiency and safety when anterograde and retrograde viral vectors that were combined with the regulatory system of gene expression were simultaneously infected as described above. In addition, efficiency of gene delivery with viral vectors is often different among various cell types or animal species [1, 13, 14].

**Fig. 1:**
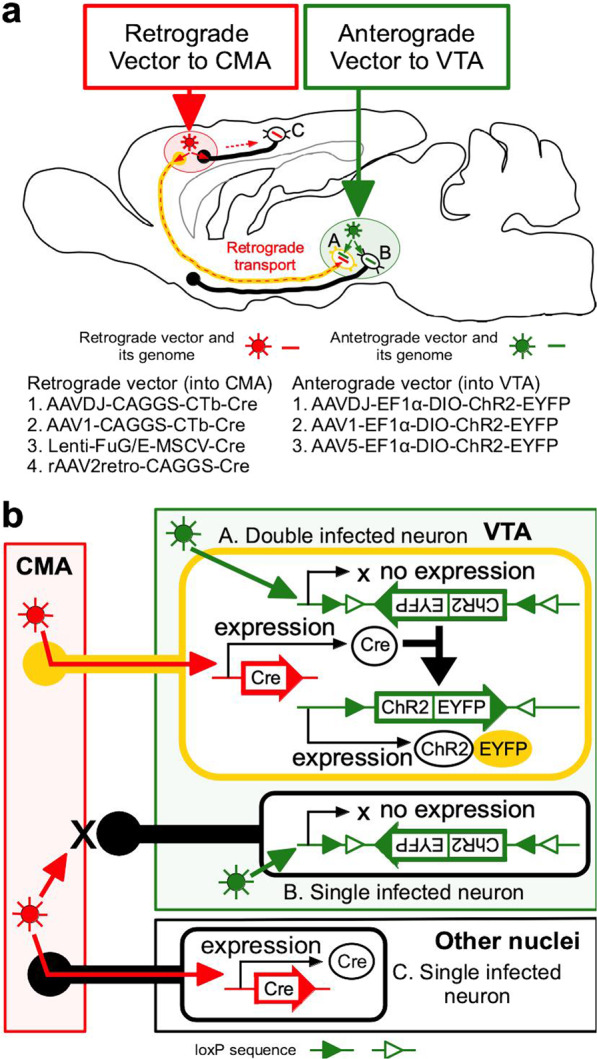
Outline of the double viral vector technology for selective manipulation of the VTA-CMA pathway. **a** In this study, we tested 4 types of retrograde vectors and 3 types of anterograde vectors. The retrograde vector that carried CAGGS or MSCV promoter and *Cre* was injected into the CMA, whereas the anterograde vector that carried EF1α promoter and *ChR2-EYFP* between loxP sequences was injected into the VTA. **b** When the neurons that express the Cre recombinase are infected with the anterograde vector, the only double infected neurons (neuron A in **a** and **b**) produce ChR2-EYFP fusion proteins. In this study, only VTA-CMA neurons were doubly infected by an anterograde vector injected into the VTA and a retrograde vector injected into the CMA and express EYFP, while other VTA neurons which were not projecting to CMA (neuron B in **a** and **b**) and neurons in other areas projecting to CMA (neuron C in **a** and **b**) are single infection and do not express EYFP. Abbreviations: ChR2 channelorhodopsin-2, CMA cortical motor area, EYFP enhanced yellow fluorescent protein, VTA ventral tegmental area.

In this study, to develop pathway-selective gene therapy in order to modulate the functions of particular pathways, we have focused on evaluating the efficiency and safety of gene transduction using double vector system that combined conventional and new vectors for enabling optogenetic control of the dopaminergic pathway from the ventral tegmental area (VTA) to the cortical motor area (CMA) in rats. The dopaminergic VTA-CMA pathway is involved in promoting the recovery after brain and spinal cord injury in the animal models as well as in motor skill learning [9, 15–18]. Here, we have tested several viral vectors that display infective tropism in dopaminergic neurons to find the optimal combination of anterograde and retrograde vectors for safe and efficient gene delivery to VTA-CMA pathway [19–30] (Fig. 1a). The viral vectors carried *Cre* or *ChR2-enhanced yellow fluorescent protein* (EYFP) between loxP sequences. Thus, only cells to which both the Cre recombinase and loxP sequences are delivered could produce ChR2-EYFP fusion proteins that can be activated by light illumination (Fig. 1b). Instead of just a marker protein, the ChR2-EYFP fusion protein was used, since a previous study reported that the transduction ability of an adeno-associated vector (AAV) is affected by its genome size [31].

## Materials and methods

### Animals, viral injections, and brain sections

Sixty-five male Wistar/ST rats, weighing 300–350 g, were used. Experiments were conducted in accordance with the guidelines of the Animal Care Institute of Laboratory Animals and approved by the Animal Care Committee of the Graduate School of Medicine, Kyoto University. All efforts were made to minimize suffering and the number of animals used in the present study was kept to a minimum. Tested viral vectors were as follows (Fig. 1a): AAVDJ (type2/type4/type5/type8/type9/avian/bovine/caprine chimera) -EF1α-DIO-ChR2-EYFP (titer, 2.5 × 10^13^ viral genomes (vg) /ml), AAV1-EF1α-DIO-ChR2-EYFP (4.0 × 10^13^ vg/ml) and AAV5-EF1α-DIO-ChR2-EYFP (1.5 × 10^13^ vg/ml) as anterograde viral vectors; AAVDJ-CAGGS-Cholera toxin B subunit (CTb)-Cre (1.1 × 10^13^ vg/ml), AAV1-CAGGS-CTb-Cre (5.3 × 10^12^ vg/ml), rAAV2retro-CAGGS-Cre (2.8 × 10^12^ vg/ml) and Lenti-FuG/E-MSCV-Cre (8.7 × 10^10^ copies/ml) as retrograde viral vectors. All the AAV and lentiviral vectors were prepared as described previously [32]. Rats were randomly assigned to each vector.　The rats were deeply anesthetized with intraperitoneal injection of a mixture of ketamine hydrochloride (48 mg/kg body weight: DAIICHI SANKYO PROPHARMA, Tokyo, Japan) and xylazine (3 mg/kg: Bayer AG, Leverkusen, Germany), fixed to a stereotaxic apparatus (SR-6R-HT, Narishige, Tokyo, Japan), and were then kept anesthetized by inhalation of 1–2% isoflurane (Pfizer, NY, USA) [33]. The anterograde viral vector was injected into bilateral VTA (0.3 µl: 5.3–5.4 mm anterior to the bregma, 2.265 mm lateral to the midline, 7.8–8.0 mm below the brain surface at 10º to midline, according to the atlas of Paxinos and Watson) with a glass micropipette attached to a manipulator (SMM-200, Narishige) and a syringe pump (the rate of 100 µl/min: Legato 130, KD Scientific Inc., MA, USA), 2–3 days after the injection of the retrograde viral vector into the bilateral CMA at two sites (each 0.5 µl: 4.0 mm anterior, 2.0 mm lateral, 1.0 – 1.1 mm deep; 2.5 mm anterior, 2.2 mm lateral, 1.0–1.1 mm deep) with a manipulator (SM-15M, Narishige). Four weeks after these injections, the rats were deeply anesthetized with an intraperitoneal injection of sodium pentobarbital (130 mg/kg: Kyoritsu Seiyaku Corporation, Tokyo, Japan), and transcardially perfused with 50 mM phosphate-buffered saline (PBS), followed by 4% (w/v) paraformaldehyde in 0.1 M sodium phosphate buffer (pH 7.4). The brains were removed, postfixed overnight at 4 °C with the same fixative and then cryoprotected successively with 10, 20, and 30% (w/v) sucrose in 0.1 M sodium phosphate buffer (pH 7.4). After the brains were divided into two hemispheric blocks at the midline, each block was cut into 40-µm-thick coronal or sagittal sections on a freezing microtome (REM-710, Yamato Koki Industrial Co., Saitama, Japan). The sections were collected serially in PBS.

### Immunoperoxidase staining for EYFP

The following incubations were performed at room temperature, and the sections were rinsed with PBS containing 0.3% (v/v) Triton-X 100 (PBS-T) [33, 34]. The sections were incubated for 30 min with a blocking solution (0.6% (v/v) H_2_O_2_, 20% (v/v) dimethyl sulfoxide in methanol) to suppress endogenous peroxidase activity. After incubation for 30 minutes with 10% (v/v) normal goat serum (NGts: S-1000, Vector Laboratories, Burlingame, CA, USA) in PBS-T, the sections were incubated overnight with 1/2,000-diluted rabbit antibody against GFP (A11122, Thermo Fisher Scientific, Waltham, MA, USA) and 1% NGts in PBS-T (PBS-Tgs). Next, the sections were incubated for 2 hours with 5 µg/ml biotinylated goat antibody against rabbit IgG (BA-1000, Vector Laboratories) in PBS-Tgs, and then for 1 hour with 1/100-diluted avidin-biotinylated peroxidase complex (PK-4000, Vector Laboratories) in PBS-T. After sufficient rinsing with PBS and 50 mM Tris-buffered saline (pH 7.6) (TBS), the sections were reacted for 30 minutes with 0.01% (w/v) diaminobenzidine, 1% (w/v) nickel ammonium sulfate and 0.0015% (v/v) H_2_O_2_ in TBS. The stained sections were mounted onto gelatin-coated glass slides, dehydrated in ethanol series, cleared in xylene, and finally coverslipped with mounting medium MX (Matsunami, Osaka, Japan). Monochrome images were taken with an optical microscope BZ-9000 (KEYENCE, Osaka, Japan). In addition, Nissl-stained sections of non-injected brains were prepared to determine the cytoarchitecture according to the atlas of Paxinos and Watson [35].

### Double immunofluorescent labeling for EYFP and TH

The sections were incubated for 30 min with 10% NGts in PBS-T, and then overnight with 1/2,000-diluted rabbit antibody against GFP and 1/500-diluted mouse antibody against tyrosine hydroxylase (TH) (T2928, Sigma, St. Louis, MO, USA) in PBS-Tgs. The sections were incubated for 2 hours with 5 µg/ml Alexa Fluor 488-conjugated goat antibody against rabbit IgG (A-11034, Thermo Fisher Scientific) and 5 µg/ml Alexa Fluor 594-conjugated goat antibody against mouse IgG (A11032, Thermo Fisher Scientific) in PBS-Tgs. After mounting on the slides, the immunostained sections were coverslipped with 50% (v/v) glycerol in TBS. Color images were taken with the optical microscope BZ-9000.

### Quantitative analysis of native and immunofluorescence for EYFP

Double infected rats were prepared by injection of rAAV2retro-CAGGS-Cre (1.4 × 10^12^ vg/ml) into CMA combined with injection of AAVDJ-EF1α-DIO-ChR2-EYFP (titer, 1.0 × 10^12^ vg/ml) or AAV5-EF1α-DIO-ChR2-EYFP (1.0 × 10^12^ vg/ml) into the VTA as described above, and were transcardially perfused 3, 6, or 12 weeks after the anterograde viral injections. The sections prepared from the double infected rats were incubated for 30 minutes with 10% NGts in PBS-T, and then overnight with 1/2,000-diluted rabbit antibody against GFP and 1/200-diluted chicken antibody against NeuN (266006, Synaptic Systems, Göttingen, Germany) in PBS-Tgs. The sections were incubated for 2 h with 5 µg/ml Alexa Fluor 568-conjugated donkey antibody against rabbit IgG (A10042, Thermo Fisher Scientific) and 5 µg/ml Alexa Fluor 647-conjugated goat antibody against chicken IgG (103-605-155, Jackson ImmunoResearch, West Grove, PA, USA) in PBS-Tgs. After mounting onto the slides, the immunostained sections were coverslipped with 50% (v/v) glycerol in TBS. Digital pseudocolor images (16-bit scale) were captured with a confocal laser-scanning microscope TCS SP8 (Leica Microsystems, Wetzler, Germany), using 63× objective lens (HC PL APO CS2, NA = 1.4), zoom factor of 0.75 and photon counting mode, as based on a previous study [36]. EYFP-native fluorescence, Alexa Fluor 568 and Alexa Fluor 647 were excited with 488-nm, 561-nm, and 633-nm laser beam and observed through a prism filter set to the bandwidth of 529–537 nm, 580–630 nm, and 657–757 nm, respectively. The laser beam strength was predetermined in a preliminary capture to avoid saturation with TetraSpeck Fluorescent Microsphere Sampler kit (T7284, Thermo Fisher Scientific). The mean intensity of native fluorescence and immunofluorescence for EYFP in individual cell bodies was measured with the software Fiji (https://fiji.sc), in which the intensity of native fluorescence and immunofluorescence for EYFP in the area of a NeuN-immunopositive cell body (enclosed within a dashed line in Fig. 7) was measured at the optical section passing through the nucleoli of the cell. The intensities were normalized with the intensities of the Fluorescent Microsphere kit.

### Statistical analysis

Sample size was determined based on previous works. Wilcoxon rank sum test (Fig. 8a, b), Shapiro-Walk normality test and Pearson correlation (Fig. 8c) were performed with the software RStudio (version 1.1a, https://www.rstudio.com).

## Results

### Efficiency of viral vector combinations for transgene delivery

**Fig. 2:**
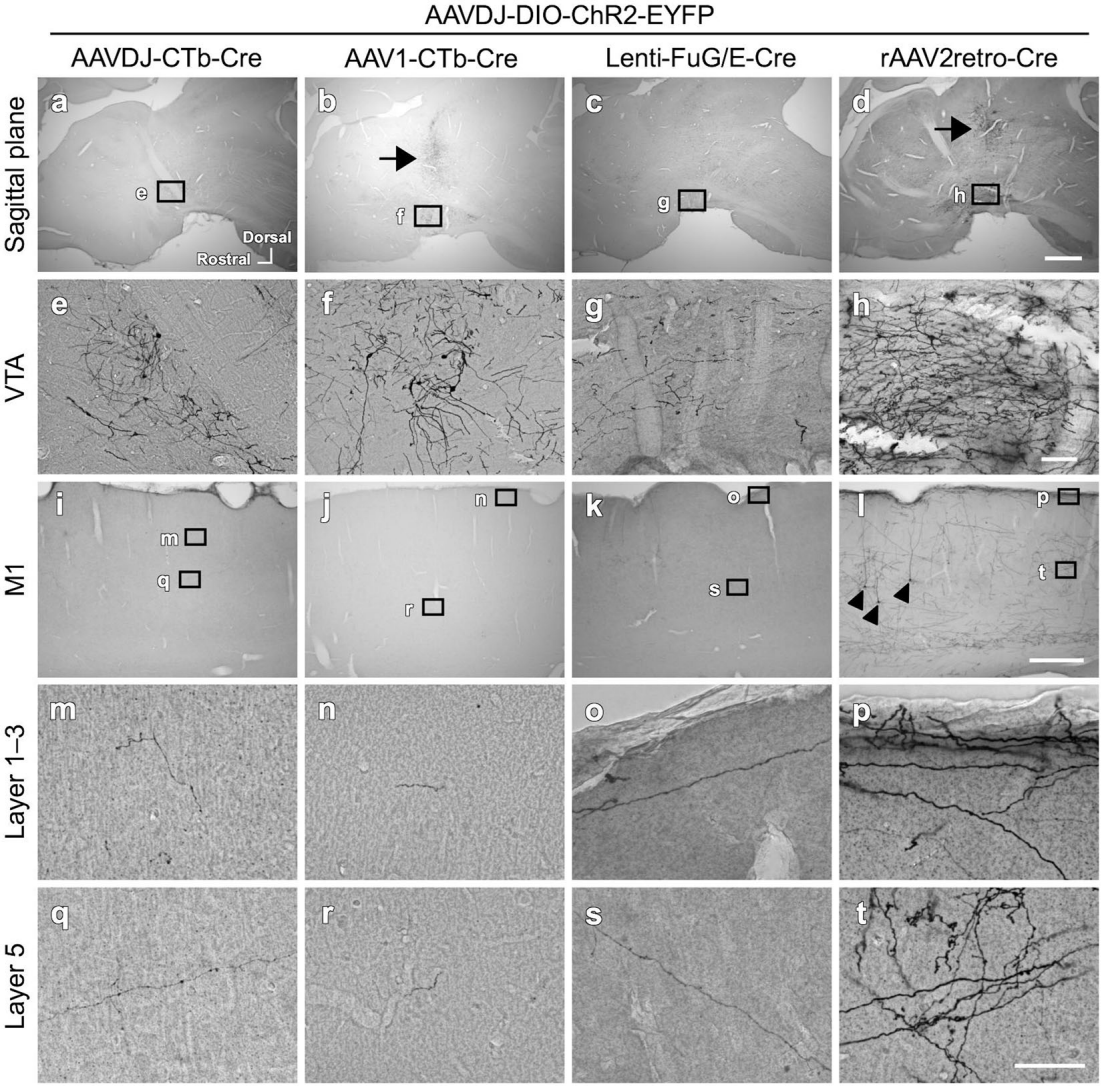
Comparison of the efficiency of 4 retrograde vectors when combined with anterograde vector AAVDJ-DIO-ChR2-EYFP. The EYFP expression in neurons double infected by anterograde AAVDJ-DIO-ChR2-EYFP and a retrograde vector was visualized with immunostaining with Ni-DAB. Each image is shown in a sagittal plane. **a**–**d** EYFP-immunopositive neurons were dominantly found in the VTA, though a few EYFP-immunopositive neurons were found along the trajectory of a glass pipet used for the injection of viral vector (arrows in **b** and **d**). **e**–**h** In the VTA, the cell bodies and neuropils of EYFP-immunopositive neurons were observed. **i**–**l** In the M1, EYFP-immunopositive axons were observed. Arrowhead in l indicates a layer 5 pyramidal neuron that exhibits EYFP immunoreactivity. **m**–**t** The EYFP-immunopositive axons in the cortical superficial layers (**m**–**p**) as well as in the deeper layers (**q**–**t**) of the cerebral cortex are shown in higher magnification images. The location of high magnification images (**m**–**t**) was taken from the black squares with corresponding letters in the lower magnification images (**i**–**l**). Scale bar = 1 mm in (**d**) (applies to **a**–**c**); 100 µm in (**h**) (applies to **e**–**g**); 500 µm in (**l**) (applies to **i**–**k**); 50 µm in (**t**) (applies to **m**–**s**).

**Fig. 3:**
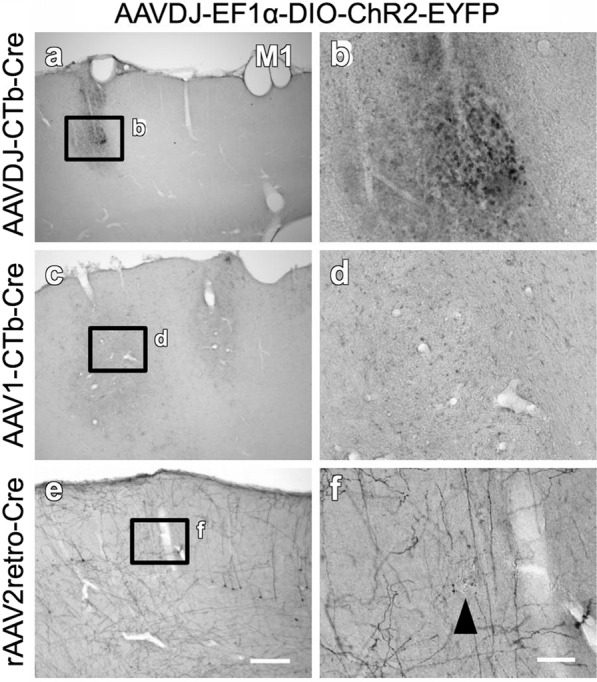
Examples of inflammatory response around the cortical injection sites. **a**, **b** The injection site of retrograde AAVDJ-CTb-Cre combined with anterograde AAVDJ exhibited perikarya-like structures. **c**, **d** The perikarya-like structures were found also around the injection site of retrograde AAV1-CTb-Cre combined with anterograde AAVDJ. **e**, **f** The injection site of retrograde rAAV2retro-CTb-Cre combined with anterograde AAVDJ did not exhibit any perikarya-like structure (arrowhead). Scale bar = 500 µm in (**e**) (applies to **a**, **c**); 100 µm in (**f**) (applies to **b**, **d**).

**Fig. 4:**
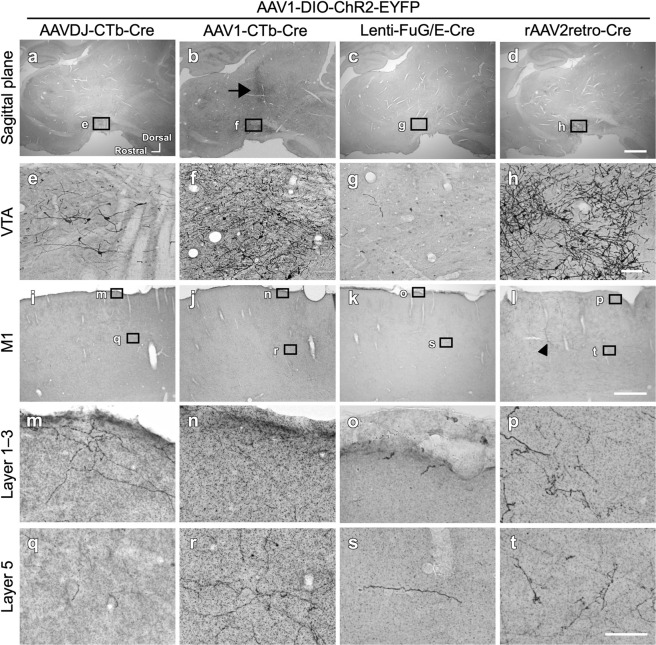
Comparison of the efficiency of 4 retrograde vectors when combined with anterograde AAV1-DIO-ChR2-EYFP. The EYFP expression in neurons with double infection by anterograde AAV1-DIO-ChR2-EYFP and a retrograde vector was visualized with immunostaining with Ni-DAB. The arrangement was the same as in Fig. 2.

**Fig. 5:**
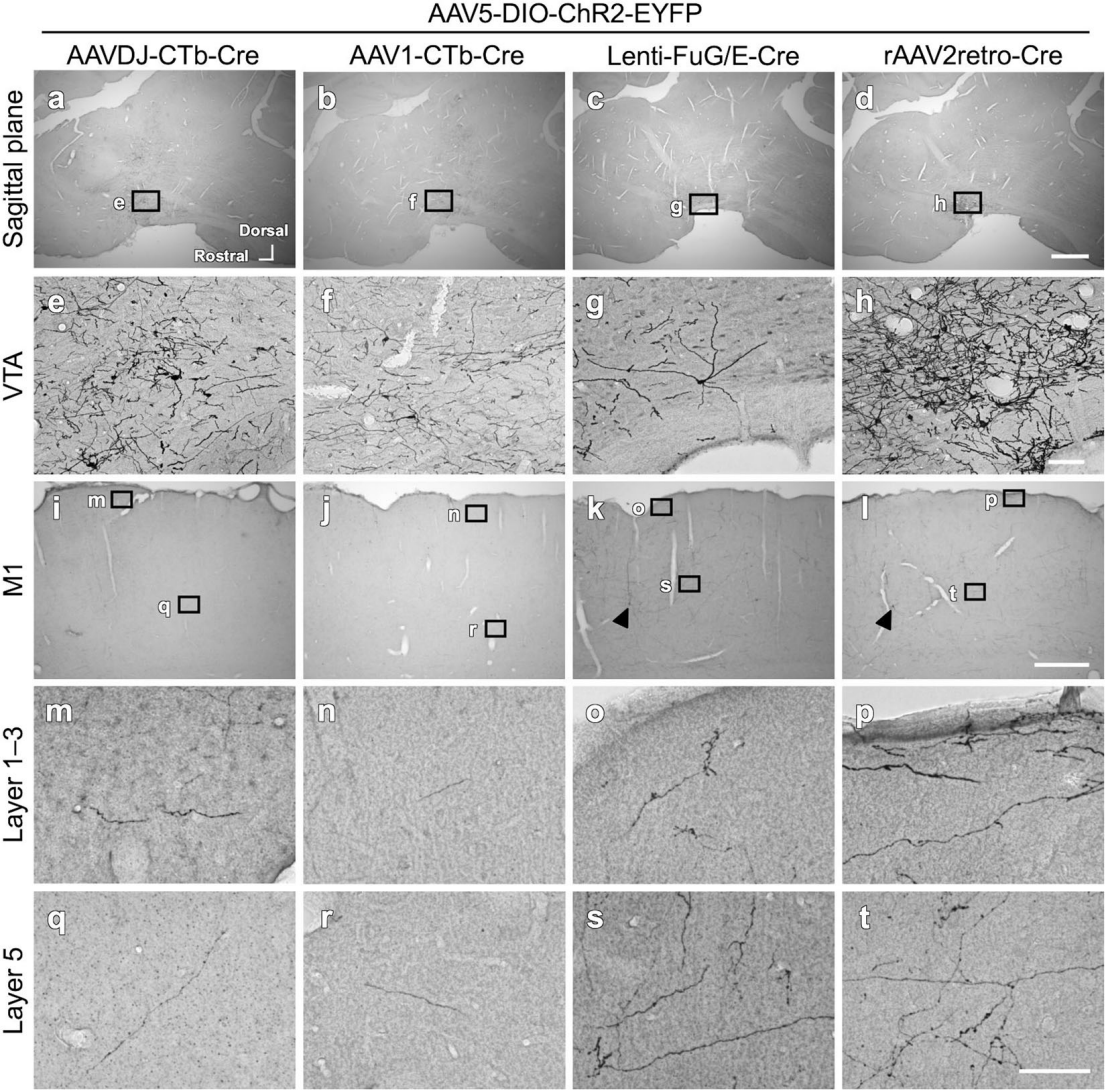
Comparison of the efficiency of 4 retrograde vectors when combined with anterograde vector AAV5-DIO-ChR2-EYFP. The EYFP expression in neurons with double infection by anterograde AAV5-DIO-ChR2-EYFP and a retrograde vector was visualized with immunostaining with Ni-DAB. The arrangement was the same as in Fig. 2.

To find the optimal combination of anterograde and retrograde viral vectors for efficient gene delivery to VTA-CMA neurons, we tested combinations of several viral vectors as shown in Fig. 1a. The mechanism for enabling pathway-selective gene expression is described in Fig. 1b. The time between the injection of the vectors and perfusion of the animals was 4 weeks. All combinations of anterograde and retrograde viral vectors had the ability to infect VTA-CMA neurons (Figs. 2, 4, and 5; Table 1). In each retrograde viral vector combined with anterograde AAVDJ, cell bodies and dendritic neuropils of EYFP-immunopositive neurons were found in the VTA (Fig. 2a–h). The cell bodies and dendritic neuropils that exhibited strong EYFP immunoreactivity were densely observed when combined with rAAV2retro (Fig. 2h), whereas they were not densely observed when combined with other retrograde vectors (Fig. 2e–g). The axonal fibers of EYFP-immunopositive neurons were also seen in the CMA (Fig. 2i–2t). The axonal fibers with sufficient EYFP immunoreactivity were densely located in both superficial and deep layers when combined with rAAV2retro (Fig. 2l, p, t), whereas the axonal density with EYFP immunoreactivity was very weak and sparse when combined with other retrograde vectors (Fig. 2i–k, m–o and q–s). EYFP immunoreactivities were found not only in a large number of VTA-CMA neurons but also in a small number of cortical layer 5 pyramidal neurons (arrowhead in Fig. 2l). This accidental exhibition of EYFP-immunoreactivity in a pyramidal neuron seems to have been caused by a retrograde uptake of AAVDJ from the corticofugal axons around the VTA. Moreover, stained perikarya-like structures were found in the injected sites of retrograde AAVDJ and AAV1 (Fig. 3a–d), whereas such stained structures were not observed in the injected site of rAAV2retro (Fig. 3e, f), indicating that the injected sites of retrograde AAVDJ-CTb and AAV1-CTb may exhibit inflammatory responses, while this is not the case for rAAV2retro. Retrograde rAAV2retro combined with either anterograde AAV1 (Fig. 4) or AAV5 (Fig. 5) also showed dense labeling of cell bodies, dendritic processes and axonal fibers with EYFP immunoreactivity in comparison with the other retrograde vectors (Table 1). In addition, the findings demonstrated differences in the staining of axonal arborizations in the CMA not only among retrograde viral vectors but also among anterograde viral vectors (Table 1). In combination with retrograde rAAV2retro, the EYFP immunoreactivity and density of stained neuropils were stronger and higher in case of anterograde AAVDJ than for anterograde AAV1 or AAV5 (Figs. 2p, t; 4p, t; 5p, t). These data suggest that retrograde rAAV2retro combined with anterograde AAVDJ may have the highest ability for gene delivery to VTA-CMA neurons among all the combinations of viral vectors where we consider the case of 4 week-survival time after injection of the vectors.

**Table 1:** The density of EYFP-immunopositive axons in the CMA compared among the different combinations of double vectors.

		Retrograde viral vector		
Anterograde viral vector	AAVDJ-CTb-Cre	AAV1-CTb-Cre	Lenti-FuG/E-Cre	AAV2retro-Cre
AAVDJ-DIO-ChR2-EYFP	++ (i)	+ (i)	+	+++++
AAV1-DIO-ChR2-EYFP	++ (i)	++ (i)	+	++++
AAV5-DIO-ChR2-EYFP	+ (i)	+ (i)	++	+++

(i), inflammation in cortical injection sites.

### Gene delivery to dopaminergic neurons

**Fig. 6:**
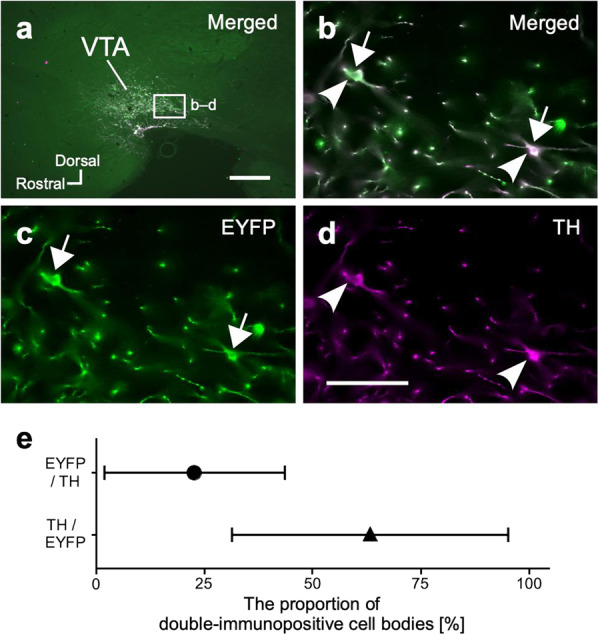
Double immunofluorescent labeling with anti-EYFP and anti-TH antibodies in the VTA. The images around the injection site of anterograde AAVDJ-DIO-ChR2-EYFP combined with rAAV2retro-CAGGS-Cre injection into the CMA are shown in sagittal planes. **a** The merged image in lower magnification clearly indicated that the immunoreactivity against EYFP and TH was dominantly found in the VTA. **b**–**d** EYFP immunoreactivity (green colored cells with white arrows) often overlapped with TH immunoreactivity (purple colored cells with white arrowheads) in the same cell bodies in the VTA (**b**). Arrows and arrowheads indicate EYFP- and TH-immunopositive cell bodies, respectively. Scale bar = 500 µm in (**a**); 50 µm in (**d**) (applies to **b**, **c**). **e** The mean proportions of double immunopositive cell bodies in 3 hemispheric brain blocks are plotted in the graph. A black circle and a triangle in the graph indicate the mean proportions of EYFP-immunopositive cell bodies among the TH-immunopositive cells (upper), and that of TH-immunopositive cell bodies among the EYFP-immunopositive cells (lower), respectively. Bars represent SEM.

To confirm whether VTA-CMA neurons infected with the combination of anterograde AAVDJ and retrograde rAAV2retro, which was found to be the best combination, contain TH immunoreactivity, a marker of dopaminergic neurons, we performed double immunofluorescent labeling for EYFP and TH in the midbrain (Fig. 6). EYFP immunoreactivity was found in the VTA where TH immunoreactivity was also abundantly observed (Fig. 6a). The EYFP immunoreactivity often overlapped with TH immunoreactivity in the same cell body and neuropil (Fig. 6b–d). The proportion of EYFP-immunopositive neurons among the TH immunopositive neurons was 22.8 ± 20.7% (mean ± SEM, *n* = 3), and that of the TH immunopositive neurons among EYFP-immunopositive cells was 63.3 ± 31.8% (mean ± SEM, *n* = 3) (Fig. 6e), indicating that a small population of dopaminergic neurons in the VTA sent axonal collaterals to the CMA, and that at least more than a half of the VTA-CMA neurons were dopaminergic neurons, even if there were a considerable number affected by false negative staining. These data suggest that the combination of anterograde AAVDJ and retrograde rAAV2retro has a strong gene delivery ability to dopaminergic neurons.

### Stability of transgene expression

**Fig. 7:**
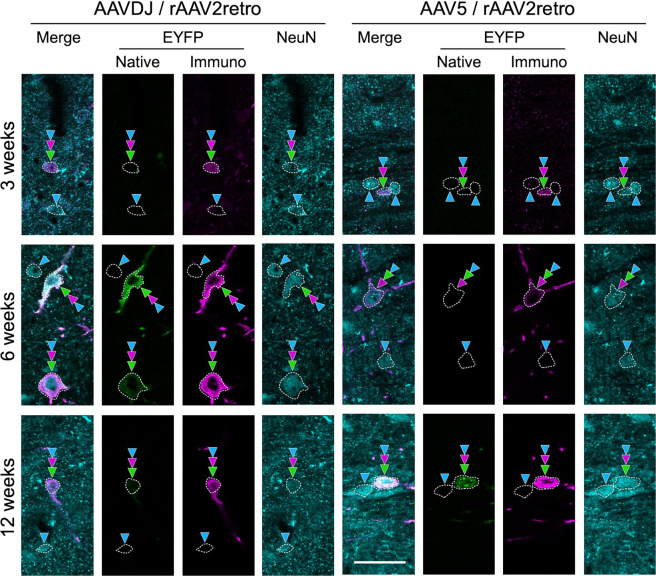
Demonstration of EYFP expression across different survival times after injection of anterograde AAVDJ/retrograde rAAV2retro versus anterograde AAV5/retrograde rAAV2retro. EYFP-native fluorescence (green color), EYFP immunofluorescence (purple color) and NeuN immunofluorescence (cyan color) in the VTA neurons are shown. Green, purple and cyan arrowheads indicate cell bodies with EYFP-native fluorescence, EYFP immunofluorescence and NeuN immunofluorescence, respectively. The intensity of native fluorescence and immunofluorescence for EYFP in the NeuN-immunopositive cell body was measured in the area enclosed with a dashed line. Scale bar = 40 µm in the merge and 12 weeks panel of AAV5/rAAV2retro (applies also to all the other panels).

**Fig. 8:**
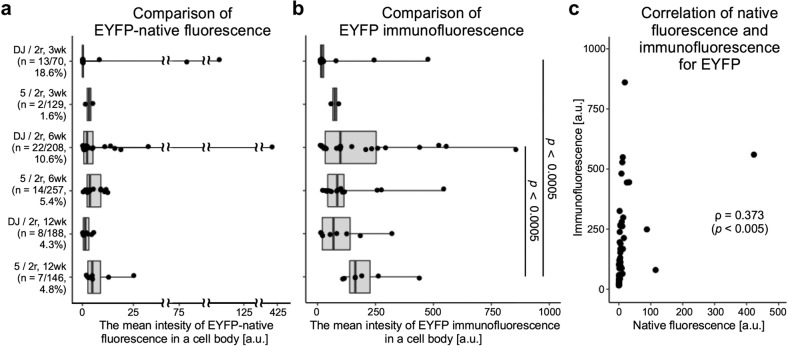
Comparison of EYFP expression across different survival times after injection of anterograde AAVDJ/retrograde rAAV2retro versus anterograde AAV5/retrograde rAAV2retro. **a** The median intensity of EYFP-native fluorescence is summarized with a box and whisker plot. Wilcoxon rank sum test did not show significant difference among all samples. The number of double infected cells counted as EYFP immunopositive cells among the NeuN-immunopositive cells is indicated along the vertical axis. **b** The median intensity of EYFP immunofluorescence is summarized with a box and whisker plot. Wilcoxon rank sum test showed significant difference between AAV5 at 12 weeks and AAVDJ at 3 or 6 weeks. **c** Correlation between intensities of native fluorescence (horizontal axis) and immunofluorescence (vertical axis) for EYFP in individual neurons shown in (**a**) and (**b**).

To investigate the stability of transgene expression in retrograde rAAV2retro combined with anterograde AAVDJ or AAV5, the intensity of native fluorescence and immunofluorescence for EYFP of individual cell bodies and the number of double infected cells were monitored up to 12 weeks (Figs. 7 and 8). The number and proportion of double infected cells counted as EYFP immunopositive cells among the NeuN-immunopositive cells is indicated on the left of Fig. 8a. Although there was some discrepancy in the intensity of native fluorescence and immunofluorescence for EYFP, they were moderately correlated with each other (Fig. 8c; ρ = 0.373, *p* < 0.005, Pearson correlation, *n* = 66). In addition, there was a clear trend that in case of the combination of anterograde AAVDJ and retrograde rAAV2retro, both intensities of native fluorescence and immunofluorescence for EYFP were already high in some infected cell bodies at 3 weeks of survival time (Figs. 7, 8a, b). An increase in the median intensity of native fluorescence and immunofluorescence for EYFP was observed at 6 weeks, whereas the percentage of the double infected cells among the NeuN-immunopositive cells was reduced to about half during each monitoring period of 3–6 weeks (18.6%–10.6 %) and 6–12 weeks (10.6%–4.3%). In contrast, in case of the combination of anterograde AAV5 and retrograde rAAV2retro, the median intensity of native fluorescence and immunofluorescence for EYFP gradually increased during the observation period (3–6 weeks and 6–12 weeks), though the percentage of infected cells was found to be slightly decreased during the period of 6–12 weeks (5.4 % to 4.8%) but higher than that of the combination of AAVDJ and rAAV2retro at 12 weeks (4.3 %). While the median intensity of EYFP-native fluorescence with AAVDJ and AAV-5 at all the survival times was not significantly different (Fig. 8a), the median intensity of EYFP immunofluorescence in case of anterograde AAV5 at 12 weeks was significantly higher than that of anterograde AAVDJ at 3 or 6 weeks (Fig. 8b). These data indicate that the combination of anterograde AAV5 and retrograde rAAV2retro may have the advantage of long-term transgene expression greater than that of anterograde AAVDJ and retrograde rAAV2retro, and it was suggested that EYFP immunofluorescence might be a more precise index to detect the difference in ability for efficient gene delivery than the EYFP-native fluorescence in the present case.

## Discussion

It is well known that many viral vectors exhibit various cell-type tropisms [13, 14]. While there are several reports on the efficiency and safety of individual viral vectors with single use [19–22, 24, 25, 27], it is not obvious whether it can explain the efficiency and safety when anterograde viral vector and retrograde viral one that were combined with the regulatory system of gene expression were simultaneously infected. Viral vectors except the CTb-fusion protein-coding AAVDJ and AAV1, which have been tested in this study, also have been reported to possess the possibility of gene delivery to dopaminergic neurons in the midbrain [19–30]. However, the optimal combination of anterograde and retrograde vectors for safe and efficient gene delivery to VTA-CMA neurons has been uncertain and whether there is an additive influence or not has also been unclear. In terms of the safety of gene delivery, retrograde AAVDJ-CTb and AAV1-CTb injected into the CMA was found to cause local damage of the cortical tissue, presumably because of an inflammatory response as the AAV capsid structure can prime an immune response of the neural tissue against a transgene product [37], even though the toxicity of AAV is generally considered to be low [1]. On the other hand, the injection sites of retrograde Lenti-FuG/E- and rAAV2retro in the CMA did not display any inflammatory responses. In terms of the efficiency of gene delivery, all cases of retrograde rAAV2retro combined with an anterograde vector were more effective in gene delivery to VTA-CMA neurons than other pairs of retrograde and anterograde vectors. Probably, these results suggest that retrograde rAAV2retro combined with an anterograde vector has the ability for safe and efficient gene delivery to VTA-CMA neurons including dopaminergic neurons, though it is not completely solved whether the efficiency of double vector systems can be explained simply by the efficiency of individual vector or there are any additive factors by combining the vectors. In addition, whether these results can be extended to other cell types in other animal species or not needs confirmation. For instance, a previous study has reported that gene delivery of Lenti-FuG/C to dopaminergic neurons in mice is low in comparison to other cell types, while it is high in macaque monkeys [27]. Moreover, recent studies have revealed that AAV1 has the ability of both anterograde and retrograde trans-synaptic transport [38, 39]. More recent one has suggested that the use of AAV1 should be limited to unidirectional circuits [39]. On the other hand, VTA dopaminergic neurons have bidirectional connections with various brain structures so that the ability of bidirectional trans-synaptic transport of AAV1 could interfere the expression of target functional protein in only VTA neurons which send axons to CMA [40–42]. Actually, ectopic EYFP-immunopositive neurons were most frequently found outside the VTA when combined with anterograde AAV1 (data not shown). Therefore, we concluded that AAV1 is not appropriate for our aim in the present study.

Gene therapy requires safe transgene expression for a long period. We also evaluated the stability of the transgene expression in double infected cells with retrograde rAAV2retro combined with anterograde AAVDJ or AAV5 along time courses. The stability of long-term transgene expression with their viral combinations is unknown, even though the single infection of anterograde AAVDJ, anterograde AAV5 or retrograde rAAV2retro has been reported to be of relatively low toxicity [24, 43, 44]. In combination with retrograde rAAV2retro, anterograde AAVDJ exhibited high transduction efficacy at the first observation point of 3 weeks. However, in the cases of anterograde AAVDJ injection, the number of double infected cells were found to largely decrease throughout the observation period, though the intensity of native fluorescence and immunofluorescence for EYFP in the cell bodies with expression was still retained. These data indicate that the combination of anterograde AAVDJ and retrograde rAAV2retro is suitable for short-term transgene expression but not for long term, though it is not clear whether this was due to the toxicity of AAJDJ or its poor ability in long term gene expression. In contrast, the combination of anterograde AAV5 and retrograde rAAV2retro increased and maintained the number of infected cells, which gradually increased the intensity of native fluorescence and immunofluorescence for EYFP in the cell bodies with expression. The intensity of EYFP immunofluorescence in the cases with anterograde AAV5 injection at 12 weeks of survival time was significantly stronger than that of anterograde AAVDJ at 3 or 6 weeks. Indeed, although the number of AAV5-infected cells was not as many as that of AAVDJ-infected cells during the earlier stage (3 and 6 weeks), the injection of higher titer AAV5 would be expected to increase the number of AAV5-infected cells, since a direct correlation between viral titer and infected cell number has previously been reported [36]. Considering these findings, AAV5 may be suitable for long-term experiments. Thus, we conclude that anterograde AAV5 and retrograde rAAV2retro is the optimal combination for safe and efficient gene delivery to VTA-CMA neurons with stable long-term transgene expression.

Our findings can be useful for pathway-selective gene therapy for neuropsychiatric disorders and traumatic neuronal injuries. In case of traumatic neuronal injuries such as stroke and spinal cord injury, new therapeutic approaches are needed for functional modulating of the transmission of descending motor pathways spared the damage [9, 45]. This approach may also be extended to the strategy of upregulating the activity of the motor cortex from subcortical centers such as the nucleus accumbens, shown in our previous study [15, 18]. Furthermore, effective pathway-specific gene delivery may be expected to improve neurodegenerative disorders such as PD. In the rat model of PD, chronic systemic levodopa treatment is known to cause the development of impulsive-like behavior [46]. If the nigrostriatal pathway can be selectively controlled by this approach, undesirable non-motor symptoms including impulsive behaviors may be avoided. In case of addiction [7], if we can selectively manipulate the mesoaccumbal pathway, we may be able to avoid possible side effects of manipulating the whole VTA that has a wide range of target areas and is involved in a variety of cognitive and non-cognitive functions. Thus, accumulating basic knowledge of pathway-specific gene delivery by double viral vector technologies is expected to realize the potential for future safe and effective therapy of neuropsychiatric disorders and traumatic neuronal injuries.
